# Sulfate but Not Thiosulfate Reduces Calculated and Measured Urinary Ionized Calcium and Supersaturation: Implications for the Treatment of Calcium Renal Stones

**DOI:** 10.1371/journal.pone.0103602

**Published:** 2014-07-25

**Authors:** Allen Rodgers, Daniel Gauvin, Samuel Edeh, Shameez Allie-Hamdulay, Graham Jackson, John C. Lieske

**Affiliations:** 1 Department of Chemistry, University of Cape Town, Cape Town, South Africa; 2 Division of Nephrology and Hypertension, Mayo Clinic, Rochester, Minnesota, United States of America; 3 Department of Laboratory Medicine and Pathology, Mayo Clinic, Rochester, Minnesota, United States of America; Emory University, United States of America

## Abstract

**Background:**

Urinary sulfate (SO_4_^2−^) and thiosulfate (S_2_O_3_^2−^) can potentially bind with calcium and decrease kidney stone risk. We modeled the effects of these species on the concentration of ionized calcium (iCa) and on supersaturation (SS) of calcium oxalate (CaOx) and calcium phosphate (CaP), and measured their *in vitro* effects on iCa and the upper limit of stability (ULM) of these salts.

**Methods:**

Urine data from 4 different types of stone patients were obtained from the Mayo Nephrology Clinic (Model 1). A second data set was obtained from healthy controls and hypercalciuric stone formers in the literature who had been treated with sodium thiosulfate (STS) (Model 2). The Joint Expert Speciation System (JESS) was used to calculate iCa and SS. In Model 1, these parameters were calculated as a function of sulfate and thiosulfate concentrations. In Model 2, data from pre- and post STS urines were analyzed. ULM and iCa were determined in human urine as a function of sulfate and thiosulfate concentrations.

**Results:**

Calculated iCa and SS values for all calcium salts decreased with increasing sulfate concentration. Thiosulfate had no effect on these parameters. In Model 2, calculated iCa and CaOx SS increased after STS treatment, but CaP SS decreased, perhaps due to a decrease in pH after STS treatment. In confirmatory *in vitro* experiments supplemental sulfate, but not thiosulfate, significantly increased the calcium needed to achieve the ULM of CaP and tended to increase the oxalate needed to reach the ULM of CaOx. Sulfate also significantly decreased iCa in human urine, while thiosulfate had no effect.

**Conclusion:**

Increasing urinary sulfate could theoretically reduce CaOx and CaP stone risk. Although STS may reduce CaP stone risk by decreasing urinary pH, it might also paradoxically increase iCa and CaOx SS. As such, STS may not be a viable treatment option for stone disease.

## Introduction

The majority of kidney stones are composed of calcium salts, mainly calcium oxalate (CaOx) and calcium phosphate (CaP) [1]. The risk of stone formation increases as the urine reaches supersaturation (SS) for these and other stone forming crystals and surpasses the threshold of spontaneous crystallization known as the upper level of metastability (ULM) [2]. SS reflects the concentration of a dissolved salt relative to the solubility of that salt in urine at body temperature [2]. Human urine is frequently supersaturated for stone-forming crystals, especially calcium oxalate [2]. Urinary SS predicts stone type as well as risk of recurrence [3]. It has been suggested that an individual’s susceptibility to stone formation is reflected by the difference between their ambient urine SS and the ULM threshold, particularly for CaP, since the greater the difference between the SS and ULM, the less likely a given crystal should spontaneously nucleate [2], [4].

Endogenous and exogenous sulfate (SO_4_^2−^) and thiosulphate (S_2_O_3_^2−^) have both been reported as affecting urinary lithogenic risk factors physiologically or physico-chemically. Sulfate is primarily produced by sulfur amino acid oxidation, which is largely eliminated by the kidney in the form of a titratable acid [5]. In general, urinary SO_4_^2−^ reflects intake of dietary proteins containing cystine and methionine [6], although 2 members of the *SLC13* family of sodium-coupled sulfate/carboxylate transporters are widely distributed in renal and gastrointestinal epithelia [7]. In particular NaS1 and NaS2 are thought largely responsible for renal sulfate reabsorption while NaC1 and NaC2 are important for citrate reabsorption, and all are under physiological and hormonal regulatory influences [7]. In addition, oxalate-sulfate exchange has recently been ascribed to the *SLC26A2* transporter found in both intestine and kidney brush border [8]. Thus, local sulfate concentrations could influence gut absorption and/or renal secretion of anions important in stone disease such as oxalate and citrate. Independent of these considerations, (SO_4_^2−^) is a divalent anion and has the capacity to bind with ionized calcium (iCa) in urine, thereby decreasing its availability for complexation with free oxalate and phosphate and concomitantly decreasing CaOx and CaP SS.

Thiosulfate (S_2_O_3_^2−^) is also a divalent anion, which therefore has the same potential capacity as sulfate for binding with Ca. Indeed, it has been proposed as a treatment for calcium nephrolithiasis following a clinical study in which urinary iCa levels and formation rates of recurrent kidney stones were significantly reduced after administration of sodium thiosulphate (STS) to a group of idiopathic calcium stone formers [9]. The authors hypothesized that formation of a calcium thiosulphate complex as a possible mechanism of the apparent beneficial effect. A subsequent study in genetic hypercalciuric stone-forming rats confirmed that STS administration reduced spontaneous stone formation but iCa did not change appreciably, and the authors expressed doubt about the formation of a calcium thiosulphate complex [10]. Recently, STS was administered to healthy controls and idiopathic hypercalciuric stone-forming patients in a pilot study [11]. Although iCa concentrations were not measured, changes in other urinary parameters did not support the hypothesis that STS could prevent stone formation.

Thus the respective roles, if any, of urinary sulfate and thiosulphate in idiopathic stone disease pathogenesis or therapy remain unresolved. Therefore, in the current study modeling calculations were performed to predict the effects of sulfate and thiosulfate on urinary iCa concentration and on CaOx and CaP SS, and to test these calculations by determining iCa and ULM in urine samples augmented with each.

## Methods

### Representative urine values

#### Mayo Clinic Stone Disease Data Base (Urine Model 1)

The Mayo Clinic Institutional Review Board approved this study. In order to obtain representative “real world” urine values for *in silico* studies, patients were identified from the Mayo Nephrology Clinic with a history of stone disease in each of the following groups (i) patients with a history of symptomatic stone passage containing a majority CaOx, without a history of enteric hyperoxaluria (n = 15); (ii) patients with a history of symptomatic stone passage containing a majority apatite or brushite (n = 13); (iii) patients with a history of cystinuria as confirmed by stone analysis or increased urinary cystine excretion (n = 10); (iv) patients with a history of symptomatic stone passage containing a majority uric acid (n = 11). These 24-hr urine samples were collected using toluene as a preservative as per the laboratory practice. The first complete urine panel in the medical record was abstracted for further analysis. The relatively high mean pH values in the uric acid and cystine stone formers indicate that these patients were likely to have been receiving some kind of therapy, but this was not regarded as a confounding factor in the modelling exercises described in this paper.

#### STS Pilot Study: Normal Controls and Hypercalciuric Stone Formers (Urine Model 2)

Urine data obtained before and after STS ingestion by a group of healthy controls and hypercalciuric stone formers [11] constituted our second urine model. The control group consisted of 5 healthcare volunteers (3 males, 2 females, mean age 33 years) with no history of urolithiasis. Participants in the patient group (4 males, 1 female, mean age 66 years) were documented stone formers with a history of hypercalciuria. Mean urinary data for all of the participants in each of the respective groups, as reported in the original study [11], were used in our calculations. Twenty four hour urinary Mg, Cl, and S_2_O_3_^2−^ excretions and urinary volumes, which were not reported in the original paper, were kindly provided by J. Asplin (Litholink Corporation, Chicago). Baseline urinary S_2_O_3_^2−^ excretions were not measured, but after STS ingestion the average excretions were 0.61 and 0.59 mM/d for controls and patients, respectively. This converts to concentrations of 2.84×10^−4^ M and 2.82×10^−4^ M, based on the mean 24 h urinary volumes of 2.15 liters and 2.09 liters respectively. However, for our modeling we used a slightly higher concentration of 5.0×10^−4 ^M for S_2_O_3_^2−^ in the respective groups for the post STS urine composition, to cover the top of the range of values reported in other studies after STS ingestion [12], [13]. For the baseline concentration of S_2_O_3_^2−^, we used a value of 1.0×10^−5^ M in accordance with the concentration previously reported for healthy controls [13].

#### Calculation of speciation concentrations and SS values

The speciation program JESS (Joint Expert Speciation System, Version 6.5) [14], [15] was used to calculate the concentration of iCa and various other urinary ionic species as well as SS of calcium oxalate and calcium phosphate salts in both models. JESS has the same limitations as those of other thermodynamic speciation programs, namely that it does not take into account kinetic phenomena and its accuracy is limited to that of its database of thermodynamic constants. However, the latter is extremely comprehensive in JESS as it includes published constants from multiple studies. Unlike other programs, it interrogates the database for each particular calculation that it is asked to perform and highlights potential anomalies for the user who is then required to select constants which are appropriate for that particular calculation. Herein lies the “Expert” aspect of the program. It can readily be appreciated that use of JESS is not routine; as such this may be regarded as one of its limitations.

In Model 1, the calculations were performed at the ambient urine sulfate concentration for each of the four classes of stone-formers, and at 10%, 200% and 500% of this value. Thiosulfate was then included in this model at low and high concentrations to examine its effect on the concentration of iCa and SS values. The low concentration of S_2_O_3_^2−^ was set at 1.0×10^−5^ M [13] while the high concentration was set at 100× this level (1.0×10^−3^ M) for modeling purposes. Importantly, this concentration is significantly in excess of levels reported after administration of STS in a pilot clinical trial [11].

In Model 2, calculation of SS values and concentrations of ionized species were performed in the baseline and post STS urine values of normal controls and hypercalciuric stone formers. In order to ascertain the effect of pH alone, the calculations were repeated using the baseline compositions, but with the pH changed to that observed in the post STS urines. Similarly, in order to assess the effect of S_2_O_3_^2−^ alone, the calculations were repeated, again using the baseline compositions and pH, but this time with the S_2_O_3_^2−^ concentration changed to that observed after STS ingestion.

#### Upper limits of metastability

ULM_CaOx_ and ULM_CaP_ were measured in human urine using a modification of the method of Asplin and colleagues [10]. Waste urine samples were obtained from stone formers and controls in whom urinary measurements for SS calculations, including sulfate, were already available. Urines were provided by the Mayo Clinic Renal Laboratory, maintained at 4°C, and studied within 24 hours of receipt. Validation data from this laboratory have previously confirmed that all analytes relevant for supersaturation calculations are stable for 7 days at this temperature. Urine pH was adjusted (using HCl or NaOH) to 5.7 (for CaOx ULM) or 6.4 (for CaP ULM). The sample was centrifuged at 3,000 rpm for 10 min, and 2 ml aliquot of the sample was transferred into a cuvette of a Cary Bio 50 UV-Visible spectrophotometer (Varian, now Agilent Technologies Inc., Santa Clara, CA). The analysis wavelength was set to 620 nm, and the threshold absorbance was set to 0.07. The cuvette was stirred at 37°C using a temperature-controlled peltier (Quantum Northwest, Liberty Lake, WA). Every 2.5 min, 5 µl of CaCl_2_ for CaP ULM or 5 µl oxalic acid solution for CaOx ULM was added to the cuvette. When the OD reading reached or exceeded the absorbance threshold, the assay was discontinued. For sulfate studies, the urine sulfate was augmented to 2 and 5 times baseline levels using a stock sodium sulfate solution. For S_2_O_3_^2−^ studies, urines were supplemented to 0.25 mM and 0.5 mM sodium thiosulfate in order to roughly cover the range seen in patients after STS ingestion [9], [11], [13]. The final concentration, either calcium or oxalate, required to reach or exceed the absorbance threshold was calculated in order to assess the ULM for CaP and CaOx, respectively using EQUIL2 rather than JESS, since previous publications on ULM used EQUIL2 [2], [4], [10]. The pH was maintained at 5.7 (CaOx) or 6.4 (CaP) for all ULM measurements. Urine samples were not re-refrigerated between measurements at baseline and after supplementation, thereby ensuring that any precipitation effects would be common to both sets of experiments.

### iCa determination

A METTLER Toledo PerfectION combination calcium electrode was used to measure iCa at a pH of 5.7 and 6.4 for the baseline and after the SO_4_^2−^ or S_2_O_3_^2−^ concentration was increased, and when the ULM for CaOx or CaP was achieved.

## Results

### Theoretical Calculations

#### Urine Model 1

**Figure 1:**
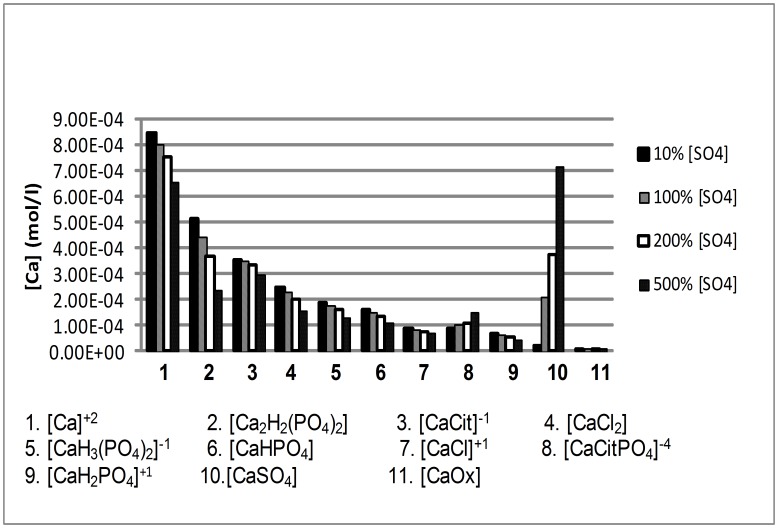
JESS-calculated concentration of calcium species as a function of [SO_4_^2−^] concentrations. Average urine values from a group of calcium oxalate stone formers were used (Table 1), and sulfate varied from baseline (100%) to as low as 10% or as high as 500% of that.

Average baseline urine concentrations for the 4 groups of stone formers are given in Table 1. JESS-calculated urinary SS and iCa values are given in **Table 2**. The concentration of all Ca species as a function of SO_4_^2−^ concentration is shown for the CaOx group in **Figure 1**. Identical trends were observed in all of the other patient groups (not shown here). SS for all Ca salts in all patient groups decreased as the concentration of SO_4_^2−^ increased (**Table 2**). It was not feasible to perform statistical comparisons of the parameters at the different concentrations of SO_4_^2–^ because the model concentrations (10%, 200%, and 500%) are theoretical; they were not determined experimentally. As such, they do not have associated experimental errors.

**Table 1:** Average urine chemistries of stone formers representing 4 different types.

Stone type	pH	Citrate(mg/dl)	Oxalate(mg/dl)	Na(mEq/l)	K(mEq/l)	Ca(mg/dl)	Phos(mg/dl)	Uric Acid(mg/dl)	Cl(mEq/l)	Creatinine(mg/dl)	Sulfate(mg/dl)	Mg(mg/dl)
**CaOx** (n = 15)	6.09	32.81	1.68	91.29	38.20	10.45	61.60	47.27	88.87	91.87	111.06	6.63
**CaP** (n = 13)	6.42	30.39	1.37	88.75	48.12	11.45	57.00	29.54	97.57	71.00	77.62	5.66
**Uric Acid** (n = 11)	5.92	35.55	2.02	89.55	48.82	10.49	66.18	51.45	98.36	107.82	141.39	6.17
**Cystine** (n = 10)	6.65	27.72	0.98	89.10	25.10	6.16	38.50	23.60	81.20	67.40	50.86	5.03

**Table 2:** JESS-calculated SS values and concentration of ionized Ca^2+^.

	COM	Bru	OCP	tCaP	HAP	Ca^2+^
CaOx Group						
10% SO_4_	3.45	1.22	348.6	12.34	3.02E+8	8.44E-4
100% SO_4_	3.15	1.13	274.8	11.36	1.89E+8	7.98E-4
200% SO_4_	2.87	1.04	219.6	7.79	1.23E+8	7.55E-4
500% SO_4_	2.25	0.86	130.8	4.66	4.40E+7	6.53E-4
10^−5^ M S_2_O_3_	3.15	1.13	274.8	11.36	1.89E+8	7.98E^−4^
10^−3^ M S_2_O_3_	3.15	1.13	274.8	11.36	1.80E+5	7.98E^−4^
CaP Group						
10% SO_4_	3.37	1.65	3073	101.9	3.31E+10	7.32E-4
100% SO_4_	3.22	1.57	2789	94.4	2.96E+10	7.13E-4
200% SO_4_	3.08	1.50	2512	87.0	2.60E+10	6.93E-4
500% SO_4_	2.70	1.32	2166	68.8	1.78E+10	6.40E-4
10^−5^ M S_2_O_3_	3.22	1.57	2789	94.4	2.96E+10	7.14E^−4^
10^−3^ M S_2_O_3_	3.22	1.58	2789	94.4	2.96E+10	7.14E^−4^
UA Group						
10% SO_4_	5.03	1.20	97.9	3.22	1.70E+7	9.10E-4
100% SO_4_	4.51	1.09	75.6	2.63	1.23E+7	8.43E-4
200% SO_4_	4.03	0.98	57.9	2.13	8.85E+6	7.82E-4
500% SO_4_	3.02	0.76	28.3	0.36	3.56E+6	6.48E-4
10^−5^ M S_2_O_3_	4.51	1.09	75.6	2.63	1.23E+7	8.42E^−4^
10^−3^ M S_2_O_3_	4.50	1.09	75.6	2.63	1.23E+7	8.42E^−4^
Cys Group						
10% SO_4_	1.20	0.91	293.9	13.0	2.67E+8	4.41E-4
100% SO_4_	1.15	0.87	263.0	11.9	2.29E+8	4.28E-4
200% SO_4_	1.10	0.83	238.4	10.7	1.96E+8	4.15E-4
500% SO_4_	0.90	0.75	179.2	8.1	1.31E+8	3.81E-4
10^−5^ M S_2_O_3_	1.15	0.87	263.0	11.9	2.29E+8	4.29E^−4^
10^−3^ M S_2_O_3_	1.15	0.87	263.0	11.9	2.29E+8	4.28E^−4^

Bru: brushite; CaOx: calcium oxalate; CaP: calcium phosphate; COM: calcium oxalate monohydrate; Cys: cystine; HAP: hydroxyapatite; OCP: octacalcium phosphate; tCaP: tri-calcium phosphate; UA: uric acid.

Results are presented as a function of [SO_4_^2−^] and [S_2_O_3_^2−^] concentrations separately for each of the 4 patient groups in **Table 1**.

The decreases in SS for all Ca salts can be accounted for by decreases in the concentration of iCa arising from complexation with SO_4_^2−^ to form CaSO_4_ (**Figure 1**). It is noted that the concentrations of all species decreased except those for CaSO_4_ and CaCitPO_4_. While the increase in the concentration of the former species is obviously due to the increase in the concentration of SO_4_^2−^, the increase in the concentration of the latter species can be accounted for by an increase in the concentration of [Cit]^3−^ (not shown here) caused by the decrease in the concentration of [CaCit] ^−1^ (due to complexation between Ca^2+^ and SO_4_^2−^) (**Figure 1**).

**Figure 2:**
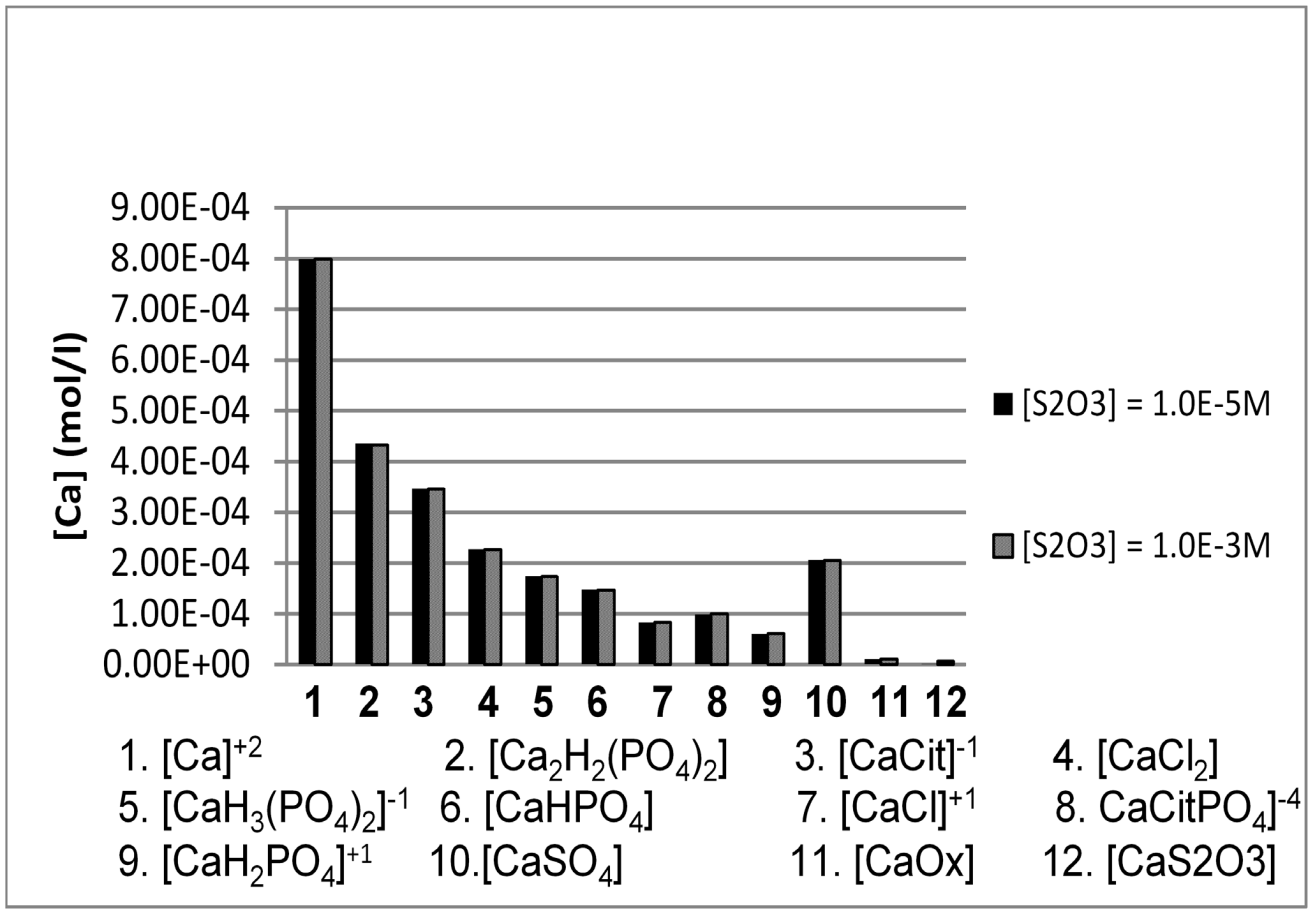
JESS-calculated concentration of calcium species as a function of [S_2_O_3_^2−^] concentrations. Average urine values from a group of calcium oxalate stone formers were used (**Table 1**), and sulfate varied from 10^−5^ M to 10^−3^ M.

Inclusion of S_2_O_3_^2−^ at low and high concentrations in the baseline model (corresponding to 100% SO_4_^2−^) had no effect on the SS value of any salt or on the concentration of iCa in any of the patient groups (**Table 2**). The stable concentration of iCa and of all other Ca species as a function of S_2_O_3_^2−^ concentrations is demonstrated in **Figure 2** for the CaOx patient group. Identical trends were observed in all of the other patient groups (not shown here).

#### Urine Model 2

Supersaturation values for various Ca salts, and concentrations of various ionized species at baseline and after administration of STS are given in the 1st and 2nd rows in **Table 3** for healthy controls and in **Table 4** for hypercalciuric stone formers. Data corresponding to other urine scenarios, discussed below, are given in the 3rd and 4th rows in both tables.

**Table 3:** JESS-calculated SS and concentrations of ionized species.

	COM	Bru	OCP	tCaP	HAP	UA	Ca^2+^	Ox^2−^	HPO_4_^−1^
Baseline (pH = 6.67) (STS = 1×10^−5^ M)	1.47	0.79	20.2	1.81	3.10E+6	0.20	3.34E-4	9.04E-5	3.71E-3
Post STS (pH = 6.08) (STS = 50×10^−5^ M)	2.12	0.70	1.72	0.17	3.16E+4	0.69	6.16E-4	7.48E-5	1.88E-3
Baseline (pH = 6.08) (STS = 1×10^−5^ M)	2.06	0.54	0.58	0.08	1.97E+3	0.69	4.72E-4	8.81E-5	1.75E-3
Baseline (pH = 6.67) (STS = 50×10^−5^ M)	1.46	0.79	20.09	1.80	3.08E+6	0.20	3.34E-4	9.05E-5	3.72E-3

Bru: brushite; CaOx: calcium oxalate; CaP: calcium phosphate; COM: calcium oxalate monohydrate; Cys: cystine; HAP: hydroxyapatite; OCP: octacalcium phosphate; sodium thiosulfate (STS); tCaP: tri-calcium phosphate; UA: uric acid.

Values were calculated using urinary values of healthy subjects at baseline and after ingestion of sodium thiosulfate [11]. SS was also re-calculated holding pH constant with increased STS, and holding STS constant with lower pH.

**Table 4:** JESS-calculated SS and concentrations of ionized species.

	COM	Bru	OCP	tCaP	HAP	UA	Ca^2+^	Ox^2−^	HPO_4_^−1^
Baseline (pH = 6.09) (STS = 1E^−5^ M)	4.57	1.59	34.51	1.56	1.15E+6	0.79	1.03E-3	8.39E-5	2.23E-3
Post STS (pH = 5.76) (STS = 50×10^−5^ M)	4.89	1.06	3.07	0.20	2.84E+4	1.64	1.36E-3	7.91E-5	1.31E-3
Baseline (pH = 5.76) (STS = 1E^−5^ M)	5.47	1.09	2.97	0.20	2.68E+4	1.46	1.26E-3	8.11E-5	1.23E-3
Baseline (pH = 6.09) (STS = 50×10^−5^ M)	4.55	1.59	34.36	1.55	1.15E+6	0.79	1.03E-3	8.40E-5	2.24E-3

Bru: brushite; CaOx: calcium oxalate; CaP: calcium phosphate; COM: calcium oxalate monohydrate; Cys: cystine; HAP: hydroxyapatite; OCP: octacalcium phosphate; sodium thiosulfate (STS); tCaP: tri-calcium phosphate; UA: uric acid.

Values were calculated using urinary values of hypercalciuric stone formers at baseline and after ingestion of sodium thiosulfate [11]. SS was also re-calculated holding pH constant with increased STS, and holding STS constant with lower pH.

Calculated COM SS increased after STS ingestion in both stone formers and controls, while SS for all the CaP salts decreased (**Tables 3** and **4**). The same trends are apparent in the CaOx and BR SS results reported by Okonkwo and colleagues [11]. These authors did not report SS values for other CaP salts, and used EQUIL2 to calculate SS. Thus results from the two studies are complimentary and consistent. Our results also show that the concentrations [Ca^2+^] in healthy controls and in stone formers increased, while those of [Ox^2−^] and [HPO_4_^2−^] decreased in post-STS urines (**Tables 3** and **4**).

Since our calculations using Model 1 demonstrated that S_2_O_3_^2−^ alone has no effect on iCa or SS of Ca salts, we concluded that the changes in SS, which were observed in the urines reported by Okonkwo et al [11] for normal controls and hypercalciuric stone formers after ingestion of STS (Model 2, **Tables 3** and **4**), must have been due to some factor other than complexation between Ca^2+^ and S_2_O_3_^2−^. A likely candidate is the statistically significant decrease in pH which occurred in both groups after ingestion of STS [11]. In order to test the hypothesis that a change in pH is the driving force behind the predicted changes in the concentration of Ca^2+^ and SS of all of the salts, we performed calculations using the baseline model in each group, but changing the pH to that which was measured after STS ingestion. Thus pH was changed from 6.67 to 6.08 and from 6.09 to 5.76 in controls and patients respectively. This shift in pH led to an increase in the [Ca^2+^] and a decrease in [HPO_4_^2−^] concentration (3rd row in **Tables 3** and **4**). The concentration of [Ox ^2−^] changed negligibly. We then performed another set of calculations, again using the baseline model, but we increased the concentration of S_2_O_3_^2−^ to that which was measured after treatment with STS. With the pH held constant, the concentration of S_2_O_3_^2−^ was increased from 1.0×10^−5^ M to 2.84×10^−4^ M in healthy controls and from 1.0×10^−5^ M to 2.8×10^−4^ M in stone formers. No changes occurred in the SS values of any of the salts or in the concentrations of any of the ionized species (4^th^ row in **Tables 3** and **4**), as had been predicted by our modeling calculations described earlier in this paper. Hence, our model confirms that the changes in SS values of all salts following ingestion of STS (**Tables 3** and **4**) are indeed due to decreases in pH in both groups, rather than to increases in the concentration of S_2_O_3_^2−^ *per se*.

**Figure 3:**
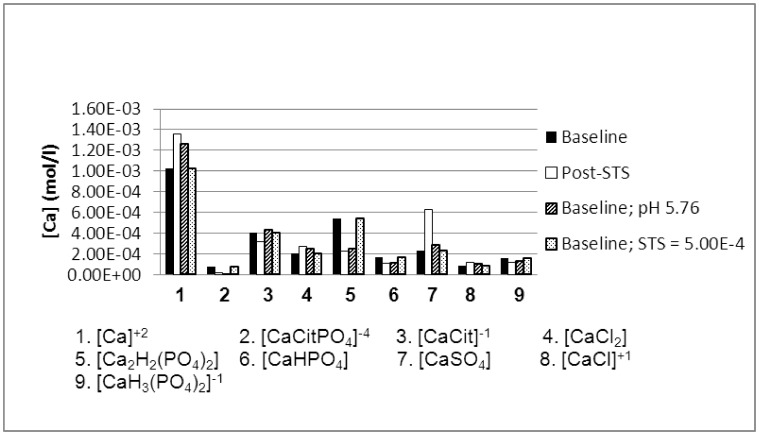
JESS-calculated concentration of calcium species as a function of thiosulfate (STS) concentration and pH. Average urine values from a group of idiopathic hypercalciurics were used before and after STS ingestion [11].

The increase in the concentration of iCa calculated in both groups following STS ingestion can be accounted for by decreases in the concentrations of several Ca species, particularly that of Ca_2_H_2_(PO_4_)_2_, which releases Ca^2+^ into the urine milieu, as shown in **Figure 3** for hypercalciuric stone formers. It has been demonstrated elsewhere that for any given urine, the concentration of this species (and others) increases as pH increases [16].

Since the concentration of Ca^2+^ increased after STS ingestion, the decreases in brushite (Bru) SS, octacalcium phosphate (OCP) SS, tri-calcium phosphate (tCaP) SS, and hydroxyapatite (HAP) SS must be accounted for by a larger fractional decrease in the concentration of [HPO_4_^2−^]. This indeed occurs (**Tables 3** and **4**), and arises because at the lower pH following STS ingestion, [HPO_4_^2−^] becomes protonated, thereby causing a decrease in its own concentration and an increase in the concentration of [H_2_PO_4_^−1^]. The concentration of [PO_4_^3−^] itself is negligible at lower pH values.

The SS of uric acid (UA) increased in both groups after ingestion of STS (**Tables 3** and **4**). Okonkwo et al found this increase to be statistically significant [11]. As with the other effects described in the preceding paragraphs, the increase in UA SS is attributed to the decrease in pH. When pH falls the concentration of H^+^ increases, thereby shifting the equilibrium towards a higher concentration of undissociated UA. This culminates in an increase in its SS level.

### Upper limits of metastability and iCa concentrations

**Figure 4:**
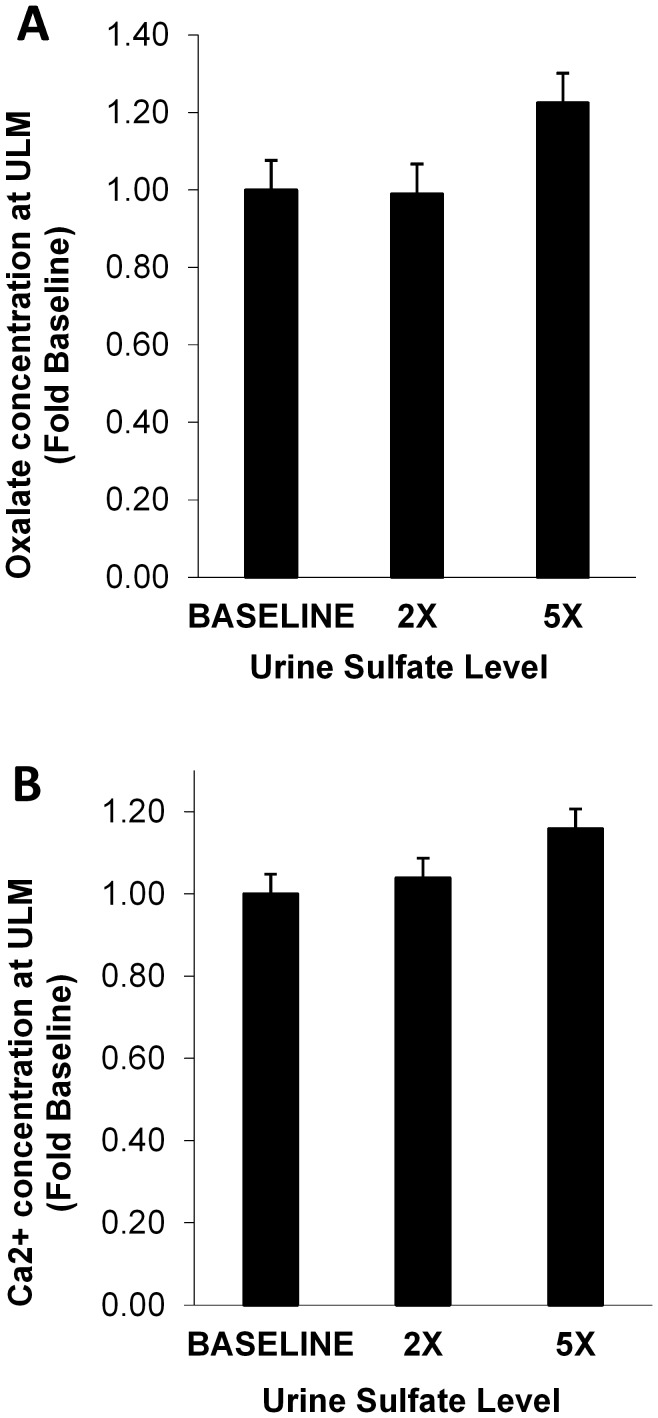
Final oxalate or calcium concentration for achieving the ULM for CaOx and CaP as a function of sulfate concentration. Urinary oxalate or calcium is expressed normalized relative to the baseline concentration in that urine sample. At 5× sulfate, 22% more oxalate (P = 0.07) and 16% more calcium (P = 0.004) was needed to reach ULM. Urine samples from 10 stone formers and 3 controls were studied. *P<0.05.

**Figure 5:**
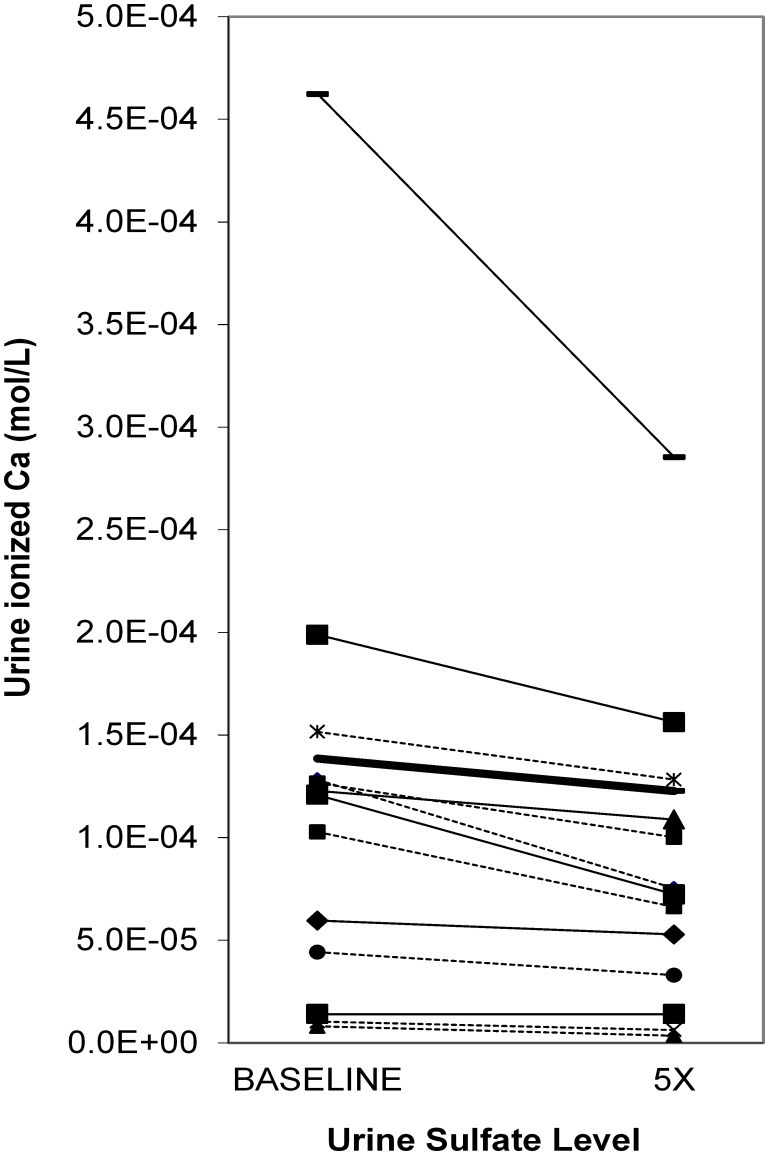
Change in concentration of urinary iCa as a function of sulfate level. Each thin line represents a different samples and the thicker line represents the average value. The pH was held constant at 5.7. Urine samples from 10 stone formers and 3 controls were studied. Overall urine calcium fell 25±15%, *P<0.001 versus baseline.

**Figure 6:**
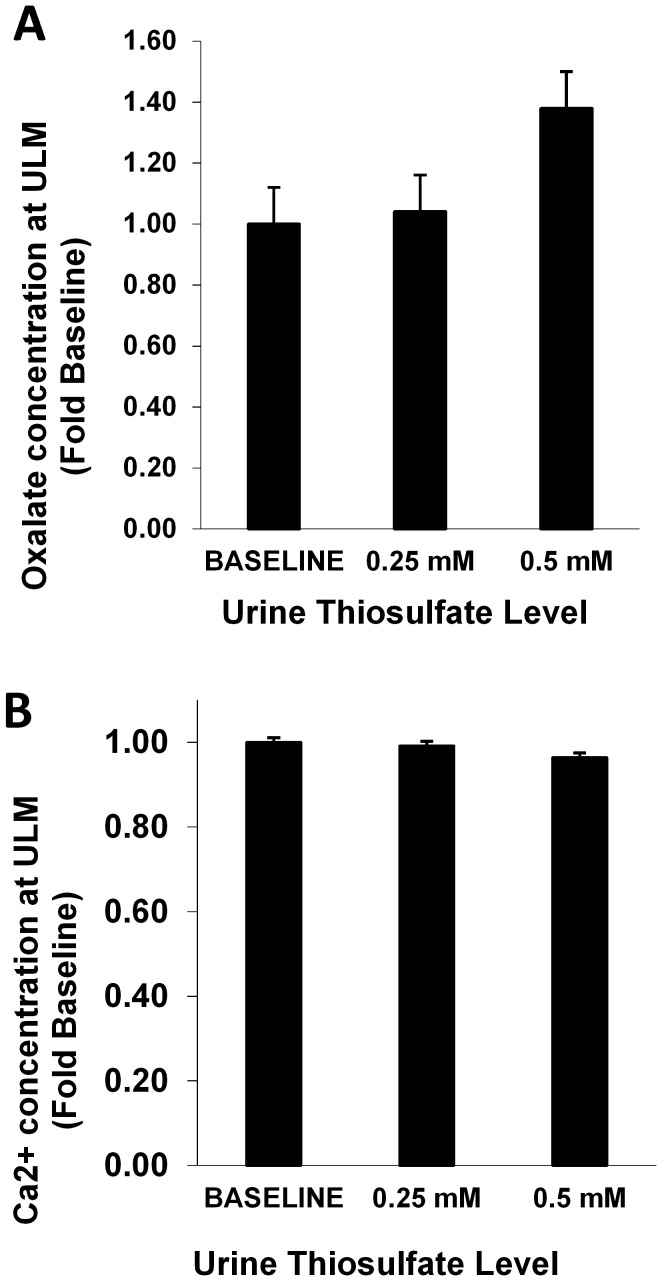
Final oxalate or calcium concentration for achieving the ULM for CaOx and CaP as a function of thiosulfate concentration. Urinary oxalate or calcium is expressed normalized relative to the baseline concentration in that urine sample. At 0.5% more oxalate (P = 0.23) and 4% less calcium (P = 0.27) was needed to reach ULM. Urine samples from 9 stone formers and 2 controls were studied.

**Figure 7:**
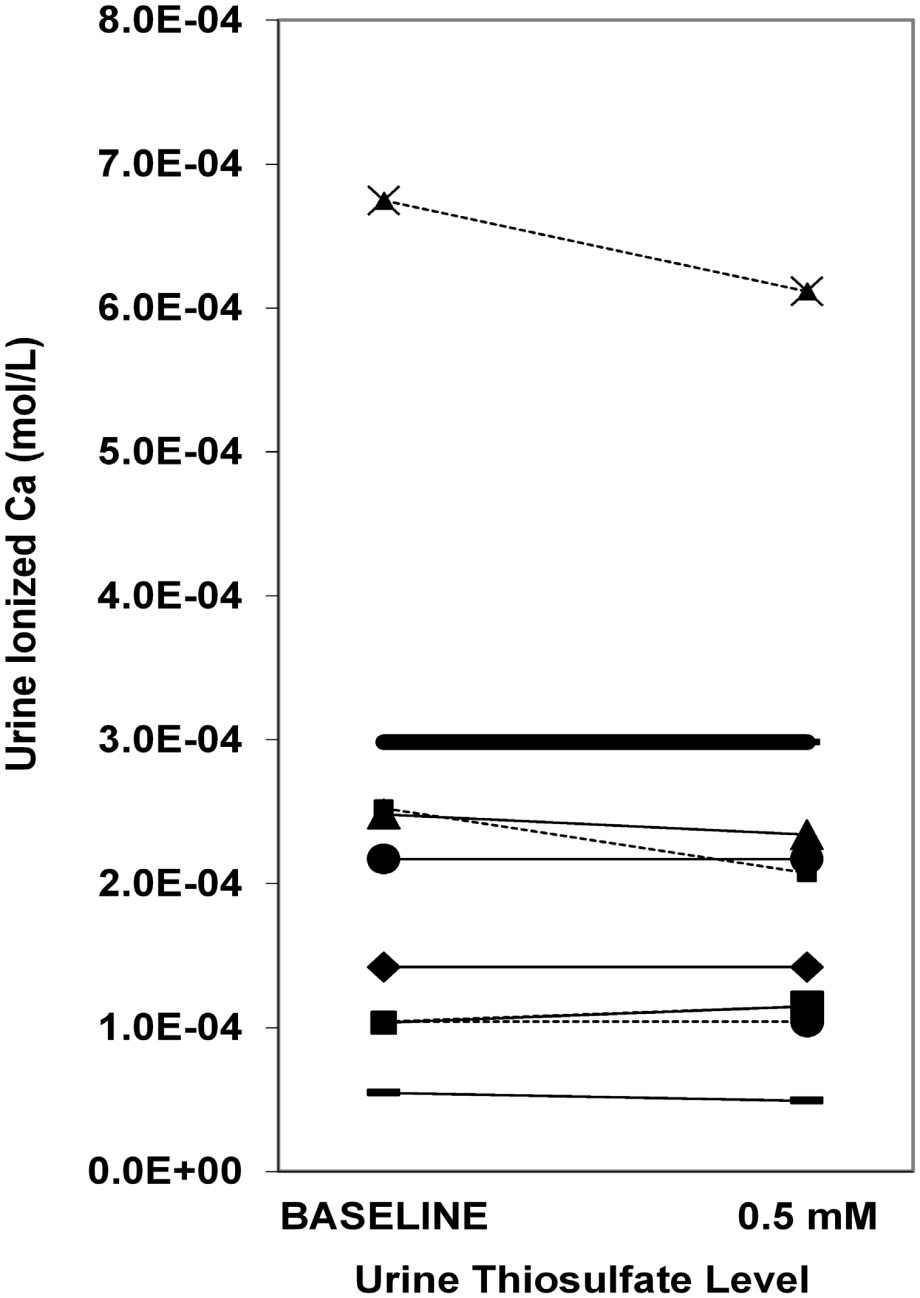
Change in concentration of urinary iCa as a function of thiosulfate level. Each thin line represents a different samples and the thicker line represents the average value. The pH was held constant at 5.7. Overall, iCa did not fall (98±8% of baseline; P = 0.41). Urine samples from 8 stone formers and 3 controls were studied.

**Figure 4** shows the amount of oxalate or calcium added to achieve ULM for CaOx and CaP respectively, as the sulfate concentration was increased to 2 or 5 times that of the baseline. Only 2 incremental increases were tested since there was no practical method to decrease urinary sulfate *in vitro*. The amount of oxalate or calcium needed to achieve the CaOx and CaP ULM tended to increase when the sulfate concentration was raised to 5 times the baseline (22%, p = 0.07 and 16%, p = 0.004 for CaOx and CaP respectively). The effect of exogenous sulfate on iCa is shown in **Figure 5** when pH was held constant at 5.7. All 13 samples showed a decrease in iCa concentration with the average iCa concentration dropping 25% from 1.21×10^−4^ to 8.87×10^−5^ mol/l (p<0.001). Similar results were observed at pH 6.4. Conversely, exogenous S_2_O_3_^2−^ did not change the amount of calcium or oxalate needed to achieve ULM or alter iCa in the 11 urine samples studied (**Figure 6**). Addition of S_2_O_3_^2−^ also did not alter iCa at pH 5.7 (**Figure 7**) or pH 6.4 (not shown).

## Discussion

Our theoretical modeling demonstrated that SO_4_^2−^ has a modest effect reducing the concentration of iCa and, concomitantly, reducing urinary SS of Ca salts. Importantly, our *in vitro* measurements confirmed the theoretical prediction of a reduction in iCa and demonstrated that SO_4_^2−^ raises the ULM for CaOx and CaP (albeit that the increase in ULM for CaOx did not quite reach statistical significance). As such, its use as a therapeutic agent in the management of Ca stone formation is worth debating.

An obvious question is whether the urinary excretion of sulfate differs between healthy controls and stone forming patients. However, data on urinary sulfate excretion in stone formers as a group are sparse. One study suggested stone formers had a higher fractional excretion of sulfate compared to controls [17]. Unfortunately, total sulfate excretion was not reported and diet was not controlled in this study. A second study of 30 matched stone formers and controls did not find a difference in total excretion [18]. In addition, a twin study suggested that urinary sulfate levels are determined largely by environment, rather than genetics [19].

Based on the results of our theoretical modeling and *in vitro* experimentation, increased urinary sulfate concentrations could reduce calcium crystal formation. However, it is not obvious how this can be effectively achieved among stone forming patients. Short term sulfate loading, whether via sulfate infusion or sulfate amino acid oxidation, is associated with enhanced urinary calcium excretion [20]. Indeed, the latter is highly correlated with urinary sulfate excretion, more so than with urea or sodium excretion, perhaps due to the variable methionine/cysteine-cystine content of dietary proteins [21]. In longer term chronic protein loading, the hypercalciuria may resolve, perhaps due to renal adaptive effects [22]. Among stone formers lower bone mineral density appears to correlate with higher urine calcium and ammonium excretion, the later presumably reflecting a greater acid load [23]. However, bone mineral density does not appear to correlate with urinary sulfate excretion. Therefore, the precise relationship between protein intake, urine sulfate, urine calcium, bone health, and kidney stone disease remains to be established.

Ammonium sulfate has been used in a small number of patients with CaP stones with reported decreases in urinary pH, CaP SS and stone rates, without changes in urinary calcium [24]. L-methionine has been used in small numbers of similar patients with CaP stones and possible infection [25]. Although much more research is needed, small case series such as these suggest that among CaP stone formers, methods to raise urinary sulfate and decrease pH may have some merit.

Conversely, our theoretical modeling and *in vitro* experimentation also demonstrated that STS *per se* has no effect on urinary iCa or on SS of Ca salts. However, our calculations showed that the decrease in urinary pH associated with STS ingestion favorably decreases SS of CaP salts, albeit that it is accompanied by an unfavorable increase in SS CaOx. Its use as a therapeutic agent in the treatment of CaP stones is therefore worth considering.

Recently STS was used in a genetic hypercalciuric CaP stone-forming rat model [10]. Stone formation dramatically decreased. Total urine calcium increased and urine pH fell. Ionized Ca was not directly measured in urine, but measurements in buffered solutions suggested that STS in concentrations up to 4 mmol/l would not change urinary iCa sufficiently to explain the beneficial effects in terms of a reduction in stone formation. The authors concluded that other mechanisms must be in play. Our data supports this assertion. For example, as pointed out by the authors themselves [10], since oxidative stress is believed to be a causative factor in kidney stone formation [26], the fact that STS is a powerful reducing agent might be important. However, we suspect that a more likely explanation for the reported decrease in CaP stone formation could be the fall in pH, with concomitant decreases in the SS of all CaP salts, as predicted by our calculations in the present study.

The potential use of STS for stone prevention was reported in 1985 [9]. In the latter study patients were given 20 mmol/day STS, which increased urine levels of S_2_O_3_^2−^ from about 0.5 to 4 mmol/day, and decreased urinary iCa from about 3.5 to 2 mmol/day [9]. In this non-controlled trial, stone recurrence rates fell from 0.98 to 0.11/year. This dose was well tolerated, and is similar to the amount used orally as secondary prevention of calciphylaxis in hemodialysis patients [27], [28] and in a few patients with nephrocalcinosis attributed to distal renal tubular acidosis [29], [30]. However, based on the theoretical and experimental findings of the present study, we find it difficult to understand how iCa could have decreased. Nor do we agree with the assertion of the authors that their observations could be attributed to the formation of a CaS_2_O_3_ complex, since we have shown in our calculations that at concentrations of S_2_O_3_^2−^ approximating those which they observed in their trial, there is no effect whatsoever on SS of CaOx and CaP salts (Model 1, **Table 2**). We are led to conclude that the decrease in stone recurrence rates which were observed might have occurred because of a decrease in urinary pH, which has been reported as being associated with STS administration [11]. Our results have shown that such a decrease in pH would lower SS of all CaP salts, possibly culminating in lower recurrence rates for this type of stone. Unfortunately, the authors of the earlier study did not report pH values, even though they stated that these were measured, nor did they indicate what type of hypercalciuric stone formers constituted their patient group [9]. We are therefore unable to test our hypothesis using the data provided in that study.

In a recent short term pilot study involving STS administration to 5 stone formers and 5 controls, investigators found decreases in urine pH and citrate and increases in urine sulfate, calcium, and ammonium [11]. The increase in urine ammonium is consistent with an acid load. Unfortunately, urinary iCa was not measured. As discussed earlier, an isolated increase in urinary sulfate is likely to modestly reduce SS of Ca salts. However, in this trial calculated CaOx SS did not fall, likely due to concurrent changes in other urine chemistries such as calcium and citrate [11]. Although the dose of STS was 20/mmol day, replicating the Yatzidis protocol [9], an average of only 0.60 mmols/d (range 0.33 to 1.22 mmols/d) was measured in the urine. Thus the amount of an oral STS load that makes it into final urine may be less than the earlier study suggested. The authors concluded that their urine chemistry results did not support the notion of STS preventing stones in rats or humans, as reported in earlier studies mentioned above [11].

The predicted ability of SO_4_^2−^ on the one hand, and the failure of S_2_O_3_^2−^ on the other hand, to exert an effect on the urinary concentration of ionized Ca^2+^ and on concomitant supersaturation values for urinary Ca salts demonstrated by our speciation modeling can be explained in terms of fundamental physicochemical principles. Firstly, since SO_4_^2−^ and S_2_O_3_^2−^ have to compete with urinary citrate to have an impact on the concentration of ionized [Ca^2+^], the respective thermodynamic binding constants (K) for the formation of [CaCit^−1^], [CaSO_4_] and [CaS_2_O_3_] need to be considered. Log K values for these complexes are respectively 3.31, 1.50 and 1.05 [15]. Comparison of these values shows that the [CaCit^−1^] complex is 63× more stable than that of the corresponding [CaSO_4_] complex and 182× more stable than that of the corresponding [CaS_2_O_3_] complex. Secondly, since the physiological concentration of SO_4_^2−^ is 1.34×10^−2^ M [31] and that of S_2_O_3_^2−^ is 1.0×10^−5^ M [13] while that of citrate is 1.90×10^−3^ M [31], it is apparent that SO_4_^2−^ would be able to compete with citrate in the formation of [CaSO_4_] only if its concentration is raised to a level which is about one order of magnitude greater than its normal value while that of S_2_O_3_^2−^ would have to be raised to several orders of magnitude greater than that of its normal value. Indeed, competition between citrate and SO_4_^2−^ is demonstrated in the formation of [CaCitPO_4_] which has been explained above in the Results section for urine model 1. Since achievement of these concentrations for S_2_O_3_^2−^ in urine is not feasible, it is extremely unlikely, if not impossible, for it to influence SS of Ca salts. This is clearly demonstrated in **Figure 2** which shows the ineffectiveness of S_2_O_3_^2−^ to exert an effect on any of the Ca species even when its concentration is significantly greater than that which has been reported after STS administration. On the other hand, achieving elevated SO_4_^2−^ concentrations is less formidable.

In conclusion, we suggest that increasing urinary sulfate concentrations may have beneficial effects for reducing SS of CaOx and CaP salts and raising their upper limits of stability, thereby reducing their risk of stone formation. Protocols for achieving the modest increases in urinary SO_4_^2−^ required for such effects are worth exploring. However, while administration of STS might achieve favorable decreases in SS of CaP salts by virtue of its ability to reduce urinary pH, the concentration required to achieve such an effect is not clinically feasible. Additionally, the concomitant increase in SS CaOx as well as the increased risk of uric acid stones at the lower pH, greatly reduces the appeal of the potential use of STS in this context. Finally, a potential complication of the acid load induced by STS is bone loss. As such, we believe that STS administration may not be a viable treatment for Ca stone disease.
